# Mental Health and Physical Activity in Women with Polycystic Ovary Syndrome: A Brief Review

**DOI:** 10.1007/s40279-014-0291-6

**Published:** 2014-11-28

**Authors:** Francesca Conte, Lauren Banting, Helena J. Teede, Nigel K. Stepto

**Affiliations:** 1 Institute of Sport, Exercise and Active Living, Victoria University, Footscray Park Campus, PO Box 14428, Melbourne, VIC 8001 Australia; 2 U.O.C. Sports Medicine, Department of Medicine, University of Padova, Padova, Italy; 3 Monash Centre for Health Research and Implementation, School of Public Health and Preventive Medicine, Monash University, Clayton, VIC 3168 Australia; 4 Diabetes and Vascular Medicine Unit, Monash Health, Clayton, VIC 3168 Australia

## Abstract

This review was designed to consider the available literature concerning mental health and physical activity in women with polycystic ovary syndrome (PCOS). A systematic approach was taken and two electronic databases (PubMed and EBSCO Research articles published between 1970 and 2013) were searched in 2013 to inform a narrative review. Inclusion criteria encompassed requirements for the research to involve a physical activity intervention and assessment of mental health outcomes in women with PCOS. Seven articles considered mental health outcomes and physical activity interventions for women with PCOS. The results demonstrated positive outcomes following physical activity intervention for health-related quality of life, depression, and anxiety. Only one paper reported the independent effects of physical activity on mental health. All other interventions included multi-factor lifestyle interventions or did not establish a control group. Physical activity is likely to be beneficial to the mental health of women with PCOS; however, more research is required to establish the nature of the relationship between physical activity and mental health outcomes.

## Key Points

**Table:** 

Management of polycystic ovary syndrome (PCOS) is complex, with strategies including diet modification, physical activity, and medication.
Physical activity intervention has been associated with positive mental health outcomes in women with PCOS.
The current literature is not sufficient to identify the independent effects of physical activity on PCOS.

## Introduction

Polycystic ovary syndrome (PCOS) is one of the most common endocrine disorders in reproductive-aged women. Depending on the diagnostic criteria used, the prevalence of PCOS varies from 6 to 18 % [1–6]. Prevalence also varies according to ethnicity [7, 8] and PCOS is also associated with factors such as body weight and lifestyle [9]. The Rotterdam criteria, which are now the internationally accepted diagnostic criteria for PCOS, require any two of the following three criteria: oligo- or anovulation, clinical and/or biochemical hyperandrogenism, and polycystic ovaries [10]. This can result in a variety of reproductive PCOS phenotypes. The reproductive features of PCOS include being the leading cause of anovulatory infertility among reproductive-aged women [6, 11]. PCOS is underpinned by insulin resistance and is associated with comorbidities including hypertension, dyslipidemia, obesity, and increased risk of metabolic syndrome and type 2 diabetes mellitus [12–15].

PCOS can also have a significant negative impact on women’s health-related quality of life (HRQoL) [16–18] and psychological function [19]. It has been reported that women with PCOS have higher levels of depression than other women [20], a finding replicated in a variety of populations [16, 19, 21]. Likewise, women with PCOS typically report higher levels of anxiety compared with healthy women [19, 20, 22]. High anxiety levels have also been reported in adolescent girls with PCOS [23]. A meta-analysis by Barry et al. [24] highlighted that psychological distress is experienced by women affected by PCOS. Compared with women with other chronic conditions, including diabetes, back pain, and arthritis, women with PCOS have been shown to have similar or better physical HRQoL, but poorer psychological HRQoL [25]. Poorer HRQoL for women with PCOS has been well established and a specific questionnaire has been developed to assess this construct in relation to the specific phenotypes of PCOS (PCOS-Q [26]). It has been suggested that the symptoms of PCOS may reduce psychological wellbeing [27] as many of the associated features of PCOS (obesity [28], infertility [29], hirsutism [30], and hormone imbalance [31]) are independently associated with poorer mental health.

In an otherwise healthy population, lifestyle modifications, in particular physical activity, are prescribed to optimize mental health conditions [32], and high levels of physical activity are associated with higher HRQoL and general vitality [33]. These improvements are also observed in people with chronic health conditions [34]. There is emerging evidence to suggest that many of the pathophysiological symptoms of PCOS can be improved by regular participation in physical activity [35, 36]. Quality of life and mental health may improve for women via the positive effects of physical activity on symptomology and comorbidity severity and/or occurrence [35]. For example, in obese populations, physical activity-induced weight reduction has been associated with improvements in depressive symptoms [36]. However, the evidence surrounding the effects of physical activity participation on mental health and HRQoL for women with PCOS is limited. Therefore, our aim is to review the available literature concerning mental health and physical activity for women with PCOS.

## Literature Search

The researchers accessed PubMed and EBSCO Research electronic databases between November and December of 2013 and searched for relevant studies between January 1970 and December 2013. The search terms included ‘polycystic ovarian syndrome’, ‘Stein-Leventhal syndrome’, ‘PCOS’, ‘exercise’, ‘physical activity’, ‘mental health’, ‘depression’, ‘anxiety’, ‘health related quality of life’, and ‘HRQoL’. Only papers written in English were considered for this review. To be included in the review, the research needed to include women with PCOS, have a focus on mental health or quality of life outcomes and prescribe or measure physical activity.

## Findings

Our search found 73 articles, but only seven papers considered mental health outcomes and implemented exercise interventions in women with PCOS (Table 1). No criteria was placed on study design; however, all studies identified reported randomized designs. To our knowledge, there is only one randomized controlled trial (RCT) with the principal aim of assessing the impact of lifestyle modification on depressive symptoms and HRQoL in women with PCOS and this study was actually a subset from a larger study [37]. In this RCT, researchers compared three different 20-week lifestyle programs for 49 overweight/obese patients: diet only, diet and aerobic exercise, or diet and combined aerobic–resistance exercise. All groups achieved similar amounts of weight loss and had similar improvements in depression and all domains of PCOS-specific HRQoL, aside from the body hair domain. However, there was no comparison of an exercise intervention with standard care or a control.

**Table 1:** Outcomes of studies assessing the influence of exercise on mental health for women with PCOS

Study	Design	*N*	Population (values are mean ± SD)	Measures	Intervention duration	Intervention(s)	Psychological outcomes
*Lifestyle interventions: diet and exercise*							
Galletly et al. [40]	RCT	28	Overweight females with PCOS HPLC: age 33.0 ± 1.2 years BMI 37.6 ± 6.4 kg/m^2^ LPHC: age 32.0 ± 1.2 years BMI 37.2 ± 6.9 kg/m^2^	HADS Rosenberg Self Esteem Rating Scale	12 weeks	Two energy-restricted diets (6,000 kJ/day) + one exercise training per week (brisk walking and aerobic and stretching exercises) 4 weeks maintenance diet HPLC diet: *n* = 14, DO: 2 LPHC diet: *n* = 14, DO: 1	Depressive symptoms decreased (*p* < 0.01) and self-esteem increased (*p* < 0.05) in HPLC group. No significant change in anxiety in either group
Thomson et al. [37]	RCT	94	Obese females with PCOS (relatively high depressive symptoms and impaired HRQoL) Age 29.3 ± 0.7 years BMI 36.1 ± 0.5 kg/m^2^	CES-D PCOSQ	20 weeks	Diet and aerobic exercise (*n* = 31, DO: 16), 5/week walking/jogging Diet and combined aerobic–resistance exercise (*n* = 33, DO: 13): 3/week walking/jogging + 2/week strength training Diet (*n* = 30, DO: 16): 6,000 kJ/day energy-restricted high-protein meal plan	All groups improved in all PCOS-specific HRQoL scores, except for body hair domain score. Clinically significant improvements in emotion, body weight and menstrual problems (*d* > 0.5)
*Exercise and medicine*							
Harris-Glocker et al. [43]	RCT	36	Obese, adolescent females with PCOS Age range: 12–18 years BMI 34.8 kg/m^2^	PCOSQ	6 months	MET group: Oral contraceptive with metformin + motivation + diet + exercise (weekly, 60-min group exercise) PBO group: Placebo + motivation + diet + exercise (weekly, 60-min group exercise)	PCOSQ total scores significantly improved in both groups, across all domains, between baseline and conclusion. No significant difference between the groups
Ladson et al. [41]	RCT	22	Obese, adolescent females with PCOS MET group: age 16.1 ± 1.5 years BMI 37.1 ± 5.8 kg/m^2^ PBO group: age 15.4 ± 1.2 years BMI 35.9 ± 6.6 kg/m^2^	PCOSQ	6 months	MET group (*n* = 11, DO: 1): metformin + 150 min/week of exercise + low-calorie diet PBO group (*n* = 11, DO: 3): placebo + 150 min/week of exercise + low-calorie diet	Improvements in overall quality of life in both groups (data not reported)
Ladson et al. [42]	RCT	114	Obese females with PCOS MET group: age 29.0 ± 4.5 years BMI 38.0 ± 7.8 kg/m^2^ PBO group: age 28.8 ± 4.6 years BMI 38.3 ± 8.0 kg/m^2^	PCOSQ	6 months	MET group (*n* *=* 55, DO: 33): metformin + low-calorie diet + 150 min/week of exercise PBO group (*n* = *59*, DO: 43): placebo + low-calorie diet + 150 min/week of exercise	Non-significant improvements in domains of quality of life (weight and emotion only) for both groups
*Other interventions*							
Nidhi et al. [39]	RCT	90	Healthy adolescent females with PCOS Exercise: age 16.2 ± 0.9 years BMI 21.2 ± 3.0 kg/m^2^ Yoga: age 16.2 ± 1.1 years BMI 20.3 ± 1.9 kg/m^2^	STAI	12 weeks	Typical exercise (*n* = 45, DO: 10): 1 h/day Traditional yoga (*n* = 45, DO: 8): 1 h/day	Both groups significantly decreased state and trait anxiety over the intervention. Yoga group decreased trait anxiety more than exercise group
Stener-Victorin et al. [38]	RCT	72	Females with PCOS Age 29.9 ± 4.4 years BMI = 28.1 ± 7.4 kg/m^2^	BSA-S MADRS PCOSQ SF-36	16 weeks	Exercise (*n* = 29): 3/week for 16 weeks, self-selected, more than 30 min Acupuncture (*n* = 28): 2/week for 2 weeks + 1/week for 14 weeks Controls (*n* = 15): oral information about exercise, no intervention	Exercise: Role physical significantly decreased, Emotions and infertility (PCOSQ) significantly increased Acupuncture: Role physical, social function, energy/vitality, general health perception, mental component, emotions (PCOSQ) significantly increased Controls: Emotions, weight, menstruation (PCOSQ) significantly increased Between-group change: Acupuncture significantly increased role physical

*BMI* body mass index, *BSA-S* Brief Scale for Anxiety, *CES-D* depression scores from the Center of Epidemiologic Studies Depression scale, *DO* drop out, *HADS* Hospital Anxiety and Depression Rating Scale, *HPLC* high protein, low carbohydrate diet condition, *HRQoL* health-related quality of life, *LPHC* low protein, high carbohydrate diet condition, *MADRS* Montgomery Asberg Depressing Rating Scale, *MET* metformin, *PBO* placebo group, *PCOS* polycystic ovary syndrome, *PCOSQ* Polycystic Ovary Syndrome Questionnaire, *RCT* randomized controlled trial, *SF-36* Short Form 36, *STAI* State-trait Anxiety Inventory

The other studies compared exercise with different forms of treatment. Stener-Victorin et al. [38] compared an acupuncture group, a control group, and an exercise group and found that all interventions increased HRQoL to a similar degree. However, there was a modest improvement in depression and anxiety score in women treated with acupuncture, compared with typical exercise and with the control group. In another study comparing a traditional exercise program with yoga among a group of adolescents [39], state and trait anxiety decreased following both interventions. The remaining research included intervention studies with exercise included as a part of a multi-factor lifestyle modification. Similar to the study by Thomson et al. [37], it is difficult to isolate the independent contribution of physical activity to the overall change observed in HRQoL, depression, and anxiety. Specifically, Galletly et al. [40] prescribed exercise in addition to different diets, while in Ladson et al. [41, 42] and Harris-Glocker et al. [43] trials exercise was complemented by metformin prescription. There was no additional benefit when exercise was combined with diet or medication (metformin) and as there was no standard care/control groups, the independent effect of the exercise in the interventions is difficult to assess.

Six of the seven research studies which have evaluated mental health and quality of life in the context of physical activity have reported positive mental health outcomes. However, the studies included in the review vary greatly in sample size and population, study design, measurement tools, concurrent therapies, and comparison group. Given this, it is difficult to draw firm conclusions about the effects of physical activity alone on the mental health outcomes of women with PCOS. The evidence supporting the association between PCOS and poorer mental health is very strong [16, 19], as is the association between PCOS and low physical activity levels [44]. Exercise is commonly advised as a positive lifestyle therapy for optimizing mental health in otherwise healthy populations [45]. However, there is limited evidence to support claims that exercise is a suitable therapy for optimizing mental health in women with PCOS. Most studies found physical activity was associated with a beneficial effect on mental health for women with PCOS. Ladson et al. [42] were the only researchers to find no significant changes on overall quality of life following an exercise program, a result that may be explained by the high levels of drop-out from the study.

## Discussion

### Exercise Therapy in PCOS and Mental Health

In a healthy population, physical activity is an effective means of managing mental health conditions [45]. This is also the case for other chronically diseased populations [46] and overweight women [47]. Therefore, it is reasonable to hypothesize that physical activity is an effective method of optimizing mental health for women with PCOS. A Cochrane review evaluating lifestyle changes in woman with PCOS [48] found significant benefits of exercise for reproductive, anthropometric, and biochemical outcomes, but few studies report a HRQoL outcome. Also, a review by Thomson et al. [35] about exercise for the treatment and management of overweight women with PCOS underlined the beneficial effects of exercise either alone or in combination with energy restriction, as a tool to improve fitness, cardiovascular, hormonal, reproductive, and psychological outcomes. It has been shown that active women with PCOS report fewer depressive symptoms than women with PCOS who are inactive, although active women with PCOS still report more depressive symptoms than active women without PCOS [49]. Many experimental studies that demonstrate positive outcomes of exercise in terms of PCOS management consider multiple lifestyle modifications, combining diet, physical activity, and motivational support sessions [50], so it is difficult to identify the specific elements of the interventions that are most effective. Additionally, satisfaction with the lifestyle changes was not assessed.

#### Existing Physical Activity Recommendations for Women with PCOS

Lifestyle modification, including physical activity, is recommended as the primary management strategy for PCOS as it reduces insulin resistance, improves metabolic and reproductive features of PCOS, and improves body image [51]. A Consensus Statement by the Androgen Excess and Polycystic Ovary Syndrome Society [52] suggested that individualized exercise programs increase compliance and suggest group or home exercise and walking as potential modes of exercise for women with PCOS. In the Australian guidelines for the management of PCOS, the authors suggest at least 150 minutes of physical activity participation each week [51]. There is a wide variety of interventions which have been tested and have primarily been designed to improve the metabolic status in women with PCOS. Most programs include some form of aerobic exercise [36, 53], with others combining aerobic and resistance exercise [54, 55] and some prescribing self-selected physical activity [37, 56]. When assessing the effect of physical activity on mental health, the setting is also very important. Previous interventions have based programs at hospitals [57], the home [54] or in a fitness facility [36]. The presence and support of other people is also likely to influence the mental health of the women exercising and it is also important to consider the effects of supervision and group exercise versus solitary exercise. The duration of the physical activity programs varies substantially, ranging from a few weeks to a year. Finally, the literature also contains a variety of different aerobic intensities and modes from moderate [38] to high intensity [36, 53] activities. The heterogeneity of exercise interventions, small sample sizes, and lack of RCTs where physical activity is assessed independently, make comparisons of outcomes difficult and specific clinical recommendations challenging with more research needed.

#### Independent Effects of Physical Activity on Mental Health for Women with PCOS

The current review provides further support that physical activity can be an important part of a lifestyle management strategy to improve the mental health and quality of life for women with PCOS. However, there is little evidence available to demonstrate the isolated effects of exercise for the psychological well-being of woman with PCOS [35]. The positive outcomes of exercise for the mental health of women with PCOS are supported by a variety of cross-sectional studies and research focusing on related outcome measures. It has been shown that a 6-month program of brisk walking can reduce significantly body image distress, despite no change in BMI in these patients [56]. Cross-sectional research also indicates that inactive women with PCOS were more likely to have mild depression [44]. Exercise may contribute to improved psychological functioning directly, and not simply via established clinical/physiological improvements (i.e., weight loss, improved fertility; [58]). It is important to understand the mechanisms by which exercise can influence the mental health of women with PCOS, as there is a plethora of evidence to suggest lower general quality of life, depression, and anxiety are more common for these women.

### Limitations of the Research

The limited research investigating mental health and exercise for women with PCOS is exploratory in nature, and serves as a starting point for more comprehensive and systematic research into the mental health outcomes of exercise for women with PCOS. The seven studies in our review included different populations in terms of BMI, age, diagnosis, and cultural background, making it difficult to compare results. The studies also prescribe different types of exercise programs which also vary in intensity, duration (from 12 weeks to 6 months), and frequency (Table 1). Small to medium sample sizes with high attrition rates (drop-out rate: 0–53 %) also make it difficult to draw firm conclusions about the effects of physical activity on mental health for women with PCOS. The designs of the studies pose the greatest limitation for assessing the effects of physical activity on mental health for women with PCOS. In the papers reviewed, exercise programs were one part of a more comprehensive lifestyle modification (diet, behavior recommendations, physical activity) or drug therapy. Most of these studies also did not include a defined control group as part of the design, making it difficult to determine whether the interventions themselves were the direct cause of the improvements in mental health. Only one study [38] included a control group in order to establish the independent effects of physical activity on the mental health of women with PCOS. In future research, it would be worthwhile to compare the effects of physical activity alongside routine care with that of routine care alone on the mental health of women with PCOS.

The studies also varied greatly in terms of participant sample. Three studies recruited adolescent females [39, 41, 43] and five studies [37, 40–43] actively recruited overweight or obese participants. Mental health pathology was not reported as an exclusion criterion in any study, and only one study [37] reported baseline depressive symptoms and impaired HRQoL among participants. These differences in samples make comparisons across studies problematic. Within studies, there is also scope to improve data reporting to allow a more comprehensive understanding of differences in treatments and over time in terms of both statistical and clinical significance.

There was also a great variety in the instruments used to assess mental health. One of the most commonly used questionnaires to assess HRQoL among women with PCOS in the literature is the Short Form-36 (SF-36) [59]; however, only Stener-Victorin et al. [38] used this measure. The PCOS-Q [26] is a specific measure of HRQoL for women with PCOS and assesses five domains: emotions, body hair, weight, infertility, and menstrual problems. This questionnaire has been shown to identify specific aspects of HRQoL that specifically affect women with PCOS [25]. This questionnaire was developed based on existing research, and interviews with women with PCOS and medical practitioners in the US [26]. Further research was conducted to establish reliability [60] and validity [25]. Additional items relating to acne were added in a modified version some years later [16] and while the questionnaire is appropriate for use in adult populations, validity in adolescence is yet to be confirmed [61]. Of the studies included in this review, five of the seven used this instrument, including two studies with adolescent populations [41, 43]. The advantage of using a PCOS-specific questionnaire compared with generic instruments is increased sensitivity for PCOS symptoms, such as increased weight or infertility [62]. Thomson et al. [37] conducted an RCT using the depression scores from the Center of Epidemiologic Studies Depression Scale (CES-D). In our review, researchers used eight different psychological tools with different outcome and scores. Although each instrument is valid and appropriate, the variety of questionnaires used to evaluate aspects of mental health makes it difficult to directly compare papers.

### Future Directions

An important consideration for future research in this field is to undertake high quality research which compares the effects of physical activity alone and typical care on the mental health of women with PCOS, across the BMI range and across diverse ethnicities. It is also important to conduct research to identify the most effective intensity, duration and types of physical activity to improve physical and mental outcomes in PCOS. Additionally, extended follow-ups may allow researchers to comment on the sustainability of mental health changes following discrete physical activity interventions. Other key research questions include how to best engage and sustain women with PCOS in physical activity programs. In future research ideally we should adopt uniform mental health assessment tools to allow comparison between studies and to inform effective management of symptoms and co-morbidities in PCOS. Finally, there is a need to identify specific barriers, preferences, and motivators for long-term participation in physical activity for women with PCOS in order to develop interventions that facilitate ongoing positive psychological and clinical outcomes. Addressing these issues requires a range of research methods to be considered and both qualitative and quantitative methods are required to gain a better understanding of the effects of physical activity on mental health for women with PCOS.

## Conclusions

Despite a sound rationale for exercise and physical activity to improve mental health in PCOS, there is limited evidence documenting the specific effects of physical activity on mental health for women with PCOS. However, the existing research does suggest that physical activity is a positive intervention for both physical and mental health for these women, with no results indicating an adverse impact. Previous research clearly indicates that women with PCOS are more likely to report higher levels of depression and anxiety and lower levels of HRQoL. More studies are now needed to understand the mechanisms underlying the relationship between mental health and physical activity for women with PCOS. There is also a need to better design studies, to use consistent measurement tools to compare different types of physical activity, intensity, and frequency of physical activity in women with PCOS in order to improve health-related outcomes including fitness, well-being, and quality of life and to inform clinical recommendations regarding the prescription of physical activity for these women.
